# A Lack of Complete Linkage Disequilibrium Between c.1236G>A and c.1129-5923C>G HapB3 Variants of *DPYD*: A Call to Revise European Pharmacogenetic Guidelines

**DOI:** 10.3390/ijms26178136

**Published:** 2025-08-22

**Authors:** Almudena Gil-Rodriguez, Sheila Recarey-Rama, Ana Fernández Montes, Ana Rodríguez-Viyuela, Francisco Barros, Angel Carracedo, Olalla Maroñas

**Affiliations:** 1 Pharmacogenomics and Drug Discovery Group (GenDeM), Health Research Institute of Santiago de Compostela (IDIS), 15706 Santiago de Compostela, Spainsheila.recarey.rama@usc.es (S.R.-R.); anarovi15@gmail.com (A.R.-V.); 2 Genomics and Bioinformatics Group, Centre for Research in Molecular Medicine and Chronic Diseases (CiMUS), University of Santiago de Compostela, 15782 Santiago de Compostela, Spain; angel.carracedo@usc.es; 3 Medical Oncology Department, University Hospital Complex of Ourense (CHUO), 32005 Ourense, Spain; afm1003@hotmail.com; 4 Centre for Biomedical Network Research on Rare Diseases (CIBERER), Instituto de Salud Carlos III, 28029 Madrid, Spain; francisco.barros@usc.es; 5 Galician Public Foundation of Genomic Medicine (FPGMX), Galician Healthcare Service (SERGAS), 15706 Santiago de Compostela, Spain; 6 Genetics Group, Health Research Institute of Santiago de Compostela (IDIS), 15706 Santiago de Compostela, Spain

**Keywords:** 5-fluorouracil, DPYD, HapB3 haplotype, linkage disequilibrium, pathogenic, regulatory agency, guideline recommendations

## Abstract

Fluoropyrimidine derivatives can cause severe toxicity in patients with DPD deficiency. Regulatory agencies, such as the European Medicines Agency (EMA), recommend pre-emptive genotyping of the HapB3 haplotype, along with other variants. Historically, the two main HapB3 variants, the benign c.1236G>A and the pathogenic c.1129-5923C>G, have been assumed to be in complete linkage disequilibrium. Recent findings contradict this assumption, questioning the reliability of the HapB3 analysis through c.1236G>A, which could directly impact patient safety. The aim of this study is to assess the linkage disequilibrium between the c.1236G>A and c.1129-5923C>G variants, with the ultimate goal of revising genotyping guidelines. A total of 46 patients already heterozygous for the c.1236G>A variant have been carefully reviewed for the c.1129-5923C>G variant. From the 46 patients analyzed, 45 maintain complete linkage disequilibrium between both variants. However, there is one patient where this linkage disequilibrium is not complete, being heterozygous for c.1236G>A and homozygous for c.1129-5923C>G. These findings challenge the validity of c.1236G>A as a surrogate marker for pathogenic variant c.1129-5923C>G. This article highlights the need for a review of the recommendations of the EMA and suggests laboratories to analyze both variants, or at least the pathogenic one, to ensure accurate therapeutic decisions.

## 1. Introduction

The fluoropyrimidine derivatives 5-fluorouracil (5-FU) and its prodrugs, capecitabine and tegafur, are a group of cytostatic drugs used in the treatment of cancers, particularly those related to the gastrointestinal tract as well as head and neck and breast cancer [1]. Fluoropyrimidine derivatives are known to cause severe toxicities in patients with partial or complete deficiency of the enzyme dihydropyrimidine dehydrogenase (DPD), encoded by the *DPYD* gene [2,3,4].

*DPYD* is a gene located on chromosome 1p21.3 and spans ~843 kb with only 3078 bp of coding regions. The gene has a coding sequence of approximately ~3 kb organized in 23 exons, ranging in length from 69 to 961 bp [5,6] and surrounded by large intronic regions with an average size of 43 kb [5]. *DPYD* exhibits extensive genetic variation, as the Genome Aggregation Database (gnomAD v2.1.1) catalogs 204 synonymous and 569 missense variants, with 40 predicted to impair enzymatic activity [7,8,9]. Also, a total of 43 haplotypes were identified across six different blocks, with all associated with severe toxicity to fluoropyrimidines in the locus-by-locus analysis [10,11,12]. Located within block *B*, the HapB3 haplotype block was initially defined in 2009 by Amstutz et al. [8] as being the most prevalent decreased function *DPYD* haplotype in European populations. The HapB3 haplotype showed an over-representation in patients with severe 5-FU toxicity [10,13,14]. Authors defined HapB3 as a combination of a synonymous SNP in the 11th exon, c.1236G>A (rs56038477), already observed in previous studies [15,16], and three intronic variants, IVS5+18G>A (rs56276561), IVS6+139G>A (rs6668296), and IVS9–51T>G (rs115632870) [8]. However, it was subsequently identified that a deep intronic mutation at c.1129-5923C>G creates a cryptic splice donor site [17]. As a result, a 44-bp fragment corresponding to nucleotides c.1129-5967 to c.1129-5924 of the 10th intron is inserted into the mature DPD mRNA [18]. This causes a frameshift, leading to a premature stop codon in the 11th exon of mature DPD mRNA, which may result in severe toxicity associated with 5-FU treatment [4,10,17,18,19].

Historically, the literature has suggested that c.1129-5923C>G and c.1236G>A are in perfect linkage disequilibrium (LD = 1) [10,20]. However, the remaining SNPs within the HapB3 haplotype do not show complete linkage disequilibrium with c.1129-5923C>G across all populations [10]. Consequently, the benign exonic variant c.1236G>A has been commonly analyzed to infer the presence of the function-altering intronic variant c.1129-5923C>G [8,10] as a tagSNP. This strategy has been beneficial in techniques such as whole exome sequencing (WES), which only covers the exonic variant c.1236G>A but not the deep intronic variant c.1129-5923C>G. In the case of HapB3, databases such as the 1000 Genomes Project report values for both r^2^ = 1.0 (a correlation coefficient that measures the relationship between SNPs; a value near 1 is indicative of a strong correlation [21]) and D’ = 1.0 (a measure of allelic association estimating historical recombination between two SNPs; values near one indicate strong correlation [21]), indicating a perfect association between the variants with no recombination events detected in the studied loci [22]. Additionally, tools like LDlink from the National Institutes of Health (NHI), which is based on referenced haplotypes from 26 different population groups in Phase 3 of the 1000 Genomes Project, also provide the same results in an efficient and user-friendly manner [23]. On the other hand, the gnomAD database shows small differences in the occurrence of both variants across the Finnish European, non-Finnish European, admixed American, and African/African American populations [24,25].

Recent data demonstrate that c.1129-5923C>G and c.1236G>A may not be in perfect linkage disequilibrium as previously assumed [26]. Turner et al. identified a case of a child who carried the benign variant in heterozygosity with no presence of the pathogenic variant, leading to the conclusion that both variants are in incomplete linkage disequilibrium [26]. The authors validated these results in 245,394 individuals of the ‘All of Us’ cohort [27]. Results showed 14 carrying the variant c.1236G>A with the c.1129-5923C>G absent [26]. These findings have significant implications, questioning the reliability of c.1236G>A as a proxy marker for the pathogenic variant. Given that several regulatory agencies currently include c.1236G>A in their recommended *DPYD* genotyping panels, these findings may require a thorough re-evaluation of existing guidelines. In April 2020 the European Medicines Agency (EMA) published a formal recommendation for genetic testing of the *DPYD* gene prior to initiating fluoropyrimidine-based chemotherapy [28]. In its technical guidance it does not specify which variants need to be analyzed [28]; however, the EMA European Public Assessment Reports (EPAR) refer to variant c.1236G>A together with the other three *DPYD* variants: c.1905+1G>A, c.1679T>G, and c.2846A>T [29]. One month later, the Spanish Agency of Medicines and Medical Devices (AEMPS) also published similar recommendations [30]. In line with this, AEMPS included the analysis of the *DPYD* gene in its pharmacogenetic biomarker database, though without specifying particular variants, issuing instead a general recommendation to perform genotyping analyses [31]. Additionally, in November 2020, the Swiss Pharmacogenomics and Personalized Therapy Group (Swissmedic) published a recommendation advocating for pre-emptive testing of four genetic variants of the *DPYD* gene in patients indicated for fluoropyrimidine-based chemotherapy [32]. AEMPS and Swissmedic align with EMA’s recommendations regarding the analysis of the same four *DPYD* variants—thus advising the c.1236G>A variant [30,32,33]. In contrast, the United States Food and Drug Administration (FDA) did not issue formal guidance until December 2022 for capecitabine and April 2024 for 5-FU injectable products, with labeling updates to include warnings about the risk of serious adverse reactions in patients with DPD deficiency [34,35]. The FDA recommends focusing on the analysis of the intronic variant c.1129-5923C>G along with three other *DPYD* variants [36].

Scientific consortia have already published recommendations associated with every *DPYD* variant. The Dutch Pharmacogenetics Working Group (DPWG) [37] and the Italian working group—comprising the Italian Association of Medical Oncology (AIOM) and the Italian Society of Pharmacology (SIF) [38]—suggest for HapB3 variants the analysis of either c.1236G>A or c.1129-5923C>G prior to fluoropyrimidine derivative prescription. In the case of heterozygous patients for any of both variants, the DPWG recommends that the standard dose be reduced by 50%, or alternatively, that fluorouracil and capecitabine be avoided altogether. However, when the pathogenic variant is presented in homozygosity, the DPWG stresses that dose adjustment cannot be based solely on the genotype [37]. In line with DPWG recommendations, the AIOM-SIF guidelines also support the use of both variants for testing purposes, recommending 75% of the standard dose for heterozygous patients and 50% of the standard dose for homozygous patients for the alternative allele [38,39]. In contrast, the French National Network of Pharmacogenetics (RNPGx) does not specifically address the HapB3 haplotype or issue specific recommendations regarding it, as its influence on DPD enzyme activity is still considered controversial [40]. The Clinical Pharmacogenetics Implementation Consortium (CPIC^®^), based on Turner’s findings, has revised its guidelines and now distinguishes these two variants separately in its recommendation tables [41]. According to these guidelines, for the pathogenic variant—regardless of whether it is present in homozygosity or heterozygosity—a 50% reduction in the initial dose of fluoropyrimidines is recommended, followed by further adjustment based on observed toxicity or, when available, therapeutic drug monitoring [41]. Notably, scientific societies such as the Spanish Society of Pharmacogenetics and Pharmacogenomics (SEFF) and the Spanish Society of Medical Oncology (SEOM) recommend the analysis of both variants but prioritize the variant c.1129-5923C>G [42]. Furthermore, the dose recommendations issued by SEFF-SEOM are in alignment with those proposed by CPIC^®^ [42]. Additionally, in June 2023, the Interterritorial Council of the National Health System (SNS) approved the Common Catalogue of Genetic and Genomic Tests, with the aim of ensuring equitable access to these tests throughout Spain. This catalogue includes the analysis of both genetic variants in HapB3 [43].

Considering recent evidence, several pharmacogenetic databases have also updated information about both variants. For instance, the Pharmacogene Variation Consortium (PharmVar) [44,45] has incorporated the information provided by Turner et al. [26] and now lists the variant c.1129-5923C>G both independently and in combination with c.1236G>A. In order to prevent incorrect functional assignment of the haplotype, PharmVar does not list the benign variant c.1236G>A separately, as it could be mistakenly interpreted as a normal-function variant [46]. Similarly, CPIC^®^ has also updated the current guidelines to reflect the same approach, listing c.1129-5923C>G separately and in combination with c.1236G>A [47].

The complexity of the HapB3 haplotype represents a challenging and multifactorial genetic element in the context of 5-FU toxicity [2,19,48,49]. In view of recent evidence that challenges the assumption of complete linkage disequilibrium between the c.1236G>A and c.1129-5923C>G variants, this study aimed to test linkage disequilibrium within the Galician population. Failure to adequately consider the available genetic evidence could have significant implications for patient stratification and therapeutic decision-making, ultimately compromising treatment safety and efficacy. Therefore, the ultimate goal is to evaluate the clinical and regulatory relevance of these results in order to propose a revision of current European guidelines regarding *DPYD* genotyping.

## 2. Results

**Figure 1:**
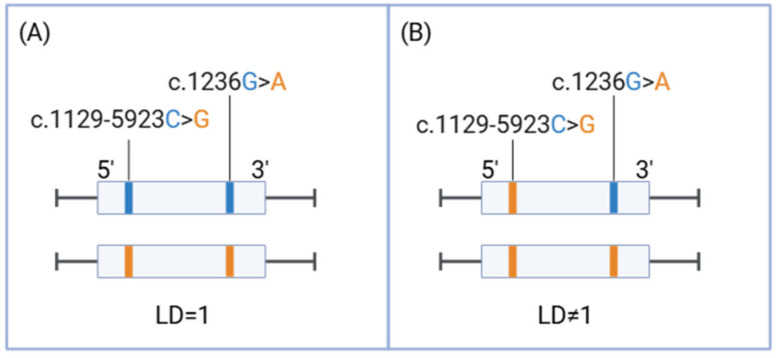
(**A**) Distribution of complete LD of 45 cases for heterozygous samples. (**B**) Distribution of the case of incomplete LD, where the variant c.1236G>A is present in heterozygosis and the variant c.1129-5923C>G is present in homozygosis. The reference allele for both variants is represented in blue, and the alternative allele (benign or pathogenic) is marked in orange.

Data from the 46 patients heterozygous for c.1236G>A were reanalyzed for the pathogenic variant c.1129-5923C>G in order to investigate the LD between both variants. Results showed that 45 patients also displayed heterozygosity for the c.1129-5923C>G variant, thus showing complete linkage disequilibrium with c.1236G>A (LD = 1). However, in the case of one patient, no complete linkage disequilibrium was observed (LD ≠ 1). This patient resulted to be heterozygous for the c.1236G>A variant and a homozygous carrier for the c.1129-5923C>G pathogenic variant (Table 1, Figure 1). The patient, a 56-year-old man, was diagnosed with diffuse-type gastric carcinoma, clinical stage cTxN2. Two cycles of neoadjuvant chemotherapy with the FLOT regimen (5-fluorouracil, leucovorin, oxaliplatin, and docetaxel) were initiated prior surgical intervention. A reduced 5-fluorouracil dose of 50% was prescribed according to the *DPYD* testing results. In the week following the first cycle, the patient developed grade 1 nausea and diarrhea considered to be treatment-related adverse events. Following the first dose of the second cycle, the patient developed grade 1 cold-induced neuropathy, which manifested between the fourth and fifth post-treatment days and was attributed to oxaliplatin. Postoperative histopathological analysis revealed a staging of pT3 pN3b R1 V1, corresponding to stage IIIC disease. Six weeks after surgery, the patient resumed oncological treatment with dose adjustments based on recommendations and tolerability (5-FU was administered at 50%, and oxaliplatin and docetaxel at 80% of the standard dose due to cumulative toxicities). Following a new multidisciplinary evaluation, the therapeutic regimen was modified to FOLFOX (calcium leucovorin (folinic acid), 5-FU, and oxaliplatin) in combination with trastuzumab, due to the patient’s genetic profile showing high levels of the Human Epidermal Growth Factor Receptor 2 (HER2) expression. It is worth highlighting that performing a co-segregation analysis in order to confirm heritability of the variants was not a viable option because the parents of the patient were deceased.

Based on the genotyping results, the 46 patients were classified as intermediate metabolizers, a phenotype that is associated with an elevated risk of severe or potentially fatal drug toxicity when treated with fluoropyrimidines [41].

Results according to the analysis of the 462 samples (197 females and 265 males) revealed complete linkage disequilibrium with c.1129-5923C>G, with none of the samples carrying the pathogenic variant (Table 1). Concerning *DPYD* results, 462 patients negative for c.1236G>A were classified as normal metabolizers with no increased risk of fluoropyrimidine-related toxicity according to guidelines. Thus, CPIC^®^ recommendations include no indications to modify dose or expected therapy [41].

## 3. Discussion

A recent study published by Turner and coworkers revealed that the benign variant c.1236G>A and the pathogenic variant c.1129-5923C>G of HapB3 were not in complete linkage disequilibrium [26]. Further co-segregation analysis revealed that the patient had inherited from the mother an allele carrying only the benign variant c.1236G>A and not the pathogenic variant c.1129-5923C>G. Authors validated these results in the ‘All of Us’ cohort [27], identifying 14 cases carrying the variant c.1236G>A, while the variant c.1129-5923C>G was absent [26]. Although no description of the dose adjustment in the patient was included, it might be assumed that, in the presence of a heterozygous result, a 50% dose reduction would have been applied in accordance with CPIC^®^ guidelines.

This finding prevents the assumption of uniform inheritance for both variants, which have been considered over the years [10]. Our study aimed to evaluate the complete linkage disequilibrium between c.1236G>A and c.1129-5923C>G in a cohort from the Galician population. A case of a patient heterozygous for c.1236G>A and homozygous for c.1129–5923C>G was identified, thus demonstrating that both variants are not in complete linkage disequilibrium. It is worth highlighting that *DPYD* pharmacogenetic results were based on the analysis of four variants proposed by regulatory agencies, EMA and AEMPS, thus c.1905+1G>A, c.1679T>G, c.2846A>T, and c.1236G>A/HapB3. According to *DPYD* genotyping results, treatment was initiated with a 50% dose reduction [41]. Considering the clinical course of the patient, no greater-than-expected toxicity was observed in response to 5-FU treatment. Adverse events recorded during the first two cycles of chemotherapy were mild (grade 1), including nausea, diarrhea, and oxaliplatin-induced neuropathy—all of which are known and common effects associated with the FLOT regimen. Subsequently, the doses of oxaliplatin and docetaxel were adjusted to 80% due to cumulative toxicities. Finally, the modification of the therapeutic regimen to FOLFOX combined with trastuzumab was driven by the HER2 overexpression observed in the molecular profile of the patient, rather than by unexpected adverse events. These findings suggest that the observed toxicity was effectively managed and remained within expected limits for this type of treatment.

It is important to highlight that incomplete linkage disequilibrium of c.1129-5923C>G and c.1236G>A can lead to different consequences in case that only the c.1236G>A is analyzed as a tagSNP. These scenarios might result in misclassification of toxicity risk, ultimately causing underdosing or overdosing of fluoropyrimidines and compromising both treatment safety and efficacy. One hypothetical scenario could be the case of patients presenting the benign variant c.1236G>A and the absence of the pathogenic variant c.1129-5923C>G. In this case, based on CPIC^®^ guidelines, a 50% reduction of the initial dose would be recommended [41]; thus, the patient would be underdosed, such as in the case presented by Turner and coworkers [26]. Another hypothetical scenario could involve the situation of non-carrier patients for the benign variant while the pathogenic variant is presented but not analyzed. In this situation, CPIC^®^ guidelines recommend starting with the standard dose in the absence of the benign variant, which could lead to treatment-related toxicity [41]. Thus, the failure in the analysis of both HapB3 variants—or at least the pathogenic variant c.1129-5923C>G—results in inaccurate therapeutic decisions based on an incomplete *DPYD* genotype. According to dose reductions, it is worth highlighting that CPIC^®^ guidelines recommend reducing by 50% the initial dose for heterozygous or homozygous carriers for analysis of either c.1236G>A and c.1129-5923C>G or c.1129-5923C>G. Thus, in the case presented in our study, the patient would have received 50% of the initial dose, although the pathogenic variant would have been analyzed. In contrast, DPWG recommends a phenotyping test along with genotyping in cases of homozygous carriers of a decreased functionality variant [37]. It is worth highlighting that these analyses have limitations that should be considered. These limitations included non-genetic factors such as age, sex, renal and hepatic function, treatments, concomitant diseases, and other genetic factors that are not included in the analysis. Concerning genetic limitations, it is important to take into consideration that no other variants of *DPYD*, apart from these five, have been analyzed. Emerging data suggest that the currently recommended 50% dose reduction by CPIC may not be necessary, as recent evidence indicates that smaller dose adjustments could achieve comparable safety and efficacy outcomes [50,51]. Another limitation of this study is that, although probes with different chemistries were used, a rare SNP could potentially interfere with the detection of both alleles; while this is very uncommon, it is not impossible and could lead to a homozygous result.

Recent *DPYD* studies conducted in the European population reflect diversity concerning the variant analyzed for the HapB3 haplotype, based on the hypothesis that both variants are in complete linkage disequilibrium. Several studies conducted in Italy [52,53,54], the United Kingdom [55], and Denmark [56,57] have focused on the c.1236G>A variant to define the HapB3 haplotype. Additionally, in the Netherlands, Henricks et al. investigated the association of *DPYD* variants with fluoropyrimidine toxicity, supporting genotype-based dose reductions, specifically considering the c.1236G>A variant in the case of HapB3 [2,48,49]. In the same line, in Spain, our group previously described *DPYD* variant frequencies in the Galician population using c.1236G>A to define HapB3, in accordance with current EMA and AEMPS recommendations [13,58]. Conversely, other studies have prioritized c.1129-5923C>G, recognizing its functional relevance. This includes the PhotoDPYD study in Spain [59], as well as studies from Greece [60] and France [61]. In this context, two recent meta-analyses conducted in 2024 have addressed these variants in patient populations. The first, conducted by Moraos et al., included 16,005 patients and focused on the c.1129-5923C>G variant [62], while the second, developed by Le Teuff et al., analyzed both variants [c.1236G>A and c.1129-5923C>G] to define the HapB3 haplotype, highlighting that both variants are in complete LD [63]. All these studies reflect differing analytical approaches alongside countries that may impact clinical interpretation and dosing decisions.

Considering the findings of the current article, together with Turner’s case, and although the frequency of the incomplete linkage disequilibrium is low, the authors consider that clinical implications are highly relevant and have to be considered.

## 4. Materials and Methods

### 4.1. Data Selection

**Figure 2:**
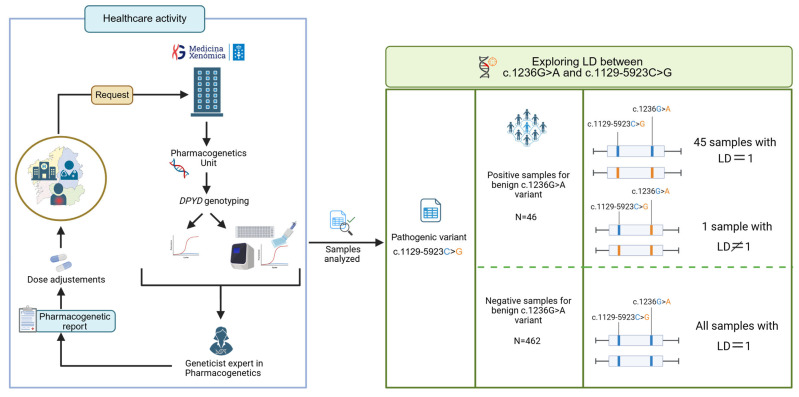
Workflow for the analysis of linkage disequilibrium data. Created with BioRender.

Samples analyzed in this report are the result of the healthcare activity performed by the Pharmacogenetics Laboratory of the Galician Public Foundation for Genomic Medicine (FPGMX) (Figure 2) [58]. In order to test the hypothesis of LD = 1 between the c.1236G>A and c.1129-5923C>G variants, a total of 46 patients positive for the c.1236G>A variant, mostly diagnosed with colorectal cancer but also with breast, rectal, or pancreatic cancer (17 females and 29 males), heterozygous for c.1236G>A, were specifically tested for the variant c.1129-5923C>G. Additionally, linkage disequilibrium was also evaluated in 462 samples that were negative for the c.1236G>A variant (Figure 2).

### 4.2. Genotyping

Following recommendations by the EMA and the AEMPS informative notes, published in April and May 2020, respectively [28], the four main *DPYD* variants, c.1905+1G>A, c.1679T>G, c.2846A>T, and c.1236G>A/HapB3, have been analyzed. Genotyping analyses were performed with real-time PCR. It is worth highlighting that, after the publication of these recommendations, the number of *DPYD* tests requested has exponentially increased [58]. Given the significant increase in the number of patients requiring genotyping of this gene, different *DPYD* analysis strategies have been developed in order to meet the growing demands of the service. In this context, *DPYD* analysis could be performed either with real-time PCR from single-tube analysis of the four aforementioned variants on the QuantStudio^TM^ 5 Real-Time PCR instrument (Applied Biosystems by Life technologies, Waltham, MA, USA) or with a customized OpenArray panel on the QuantStudio^TM^ 12K Flex (Applied Biosystems by Life technologies, Waltham, MA, USA). This array, specifically designed in our laboratory, includes, apart from the four variants, the pathogenic variant c.1129-5923C>G. Real-time PCR analyses were performed following protocols recommended by the commercial supplier [64]. A total of 7 μL of genotyping master mix and 7 μL of genomic DNA at a concentration of 25 ng/μL were mixed. Subsequently, a total volume of 5.5 μL of the mixture was transferred to the array. The PCR cycling conditions were as follows: pre-PCR hold stage at 93 °C for 10 min, followed by a PCR stage that is composed of a step of 50 cycles at 95 °C for 45 s, a step at 94 °C for 13 s, and a step at 53.5 °C for 2 min and 14 s; finally, a pre-PCR hold at 25 °C for 2 min. Negative and positive controls were used in the reactions. Samples presented in the current article have been analyzed with the customized OpenArray panel on the QuantStudio^TM^ 12K Flex (Applied Biosystems by Life technologies, Waltham, MA, USA) containing the variant c.1129-5923C>G. In the concrete case of non-linkage disequilibrium observed, the result has been confirmed using an alternative method, rhAMP^TM^ probes (Integrated DNA Technologies (IDT), Coralville, IA, USA) [65]. The rhAMP^TM^ assay was analyzed following the protocol recommended by the commercial supplier on the QuantStudio^TM^ 5 Real-Time PCR (Applied Biosystems by Life technologies, Waltham, MA, USA). These assays were used with a final reaction volume of 10 µL consisting of 5.3 µL of genotyping master mix, 0.5 µL of assay probe, 2.2 µL of nuclease-free water, and finally, 2 µL of genomic DNA at a concentration of 2.5 ng/μL. The PCR cycling conditions were as follows: enzyme activation at 95 °C for 10 min, followed by 40 cycles of a denaturation step at 95 °C for 10 s, an annealing step at 60 °C for 30 s, an extension step at 68 °C for 20 s, and finally a heat inactivation step at 99.9 °C for 15 min. Negative and positive controls were also used in this case. Interpretation of results was performed with the QuantStudio TM Design & Analysis Software v1.5.2 (ThermoFisher Scientific, Waltham, MA, USA).

## 5. Conclusions

Our findings, along with those by Turner et al., compromise the reliability of c.1236G>A as a surrogate marker for detecting the pathogenic variant c.1129-5923C>G. Therefore, authors recommend to revise current recommendations published by EMA and AEMPS concerning pharmacogenetic analysis in the gene *DPYD* in order to ensure that the pathogenic variant c.1129-5923C>G is analyzed. Additionally, authors would recommend that laboratories exercise caution and analyze both *DPYD* variants, or at least the pathogenic variant.

## Figures and Tables

**Table 1:** Baseline characteristics of patients.

Number of Patients	c.1236G>A Positive	c.1236G>A Negative
Sex (N, %)		
Male	29 (63.04%)	265 (57.36%)
Female	17 (36.96%)	197 (42.64%)
Age (mean ± SD)	68.78 ± 10.75	ND
Genotyping results (N, %):		
c.1129-5923C>G *		
C/G	45 (97.83%)	462 (100%)
G/G	1 (2.17%) *	---
c.1236G>A		
G/A	46 (100%)	462 (100%)

* Pathogenic variant. ND: no data available.

## Data Availability

The data analyzed in this study are subject to the following licenses/restrictions: Data were derived from clinical practice. Requests to access these datasets should be directed to Olalla Maroñas, olalla.maronas@usc.es.

## Abbreviations

The following abbreviations are used in this manuscript:

5-FU	5-fluorouracil
DPD	Dihydropyrimidine dehydrogenase
SNPs	Single nucleotide polymorphisms
LD	Linkage disequilibrium
WES	Whole exome sequencing
PharmVar	Pharmacogene Variation Consortium
CPIC^®^	Clinical Pharmacogenetics Implementation Consortium
EMA	European Medicine Agency
EPAR	European Public Assessment Reports
AEMPS	Spanish Agency for Medicines and Health Products
Swissmedic	Swiss Agency for Therapeutic Products
FDA	Food and Drug Administration
DPWG	Dutch Pharmacogenetics Working Group
AIOM	Italian Association of Medical Oncology
SIF	Italian Society of Pharmacology
RNPGx	French National Network of Pharmacogenetics
SEFF/SEOM	Pharmacogenomics Society and the Spanish Society of Medical Oncology
FPGMX	Pharmacogenetic Unit of the Public Foundation of Genomic Medicine
HER2	Human Epidermal Growth Factor Receptor 2
